# CFD Development of a Silica Membrane Reactor during HI Decomposition Reaction Coupling with CO_2_ Methanation at Sulfur–Iodine Cycle

**DOI:** 10.3390/nano12050824

**Published:** 2022-02-28

**Authors:** Milad Mohammad Alinejad, Kamran Ghasemzadeh, Adolfo Iulianelli, Simona Liguori, Milad Ghahremani

**Affiliations:** 1 Faculty of Chemical Engineering, Urmia University of Technology, Urmia 5756151818, Iran; milad.mohammadalinejad@gmail.com (M.M.A.); milad.gharemani@uut.ac.ir (M.G.); 2 Institute on Membrane Technology of the Italian National Research Council (CNR-ITM), Via P. Bucci Cubo 17/C, 87036 Rende, CS, Italy; 3 Department of Chemical and Biomolecular Engineering, Clarkson University, Potsdam, NY 13699, USA; sliguori@clarkson.edu

**Keywords:** silica membrane reactor, HI decomposition process, CO_2_ methanation, CFD modeling, hydrogen generation

## Abstract

In this work, a novel structure of a hydrogen-membrane reactor coupling HI decomposition and CO_2_ methanation was proposed, and it was based on the adoption of silica membranes instead of metallic, according to their ever more consistent utilization as nanomaterial for hydrogen separation/purification. A 2D model was built up and the effects of feed flow rate, sweep gas flow rate and reaction pressure were examined by CFD simulation. This work well proves the feasibility and advantage of the membrane reactor that integrates HI decomposition and CO_2_ methanation reactions. Indeed, two membrane reactor systems were compared: on one hand, a simple membrane reactor without proceeding towards any CO_2_ methanation reaction; on the other hand, a membrane reactor coupling the HI decomposition with the CO_2_ methanation reaction. The simulations demonstrated that the hydrogen recovery in the first membrane reactor was higher than the methanation membrane reactor. This was due to the consumption of hydrogen during the CO_2_ methanation reaction, occurring in the permeate side of the second membrane reactor system, which lowered the amount of hydrogen recovered in the outlet streams. After model validation, this theoretical study allows one to evaluate the effect of different operating parameters on the performance of both the membrane reactors, such as the pressure variation between 1 and 5 bar, the feed flow rate between 10 and 50 mm^3^/s and the sweep gas flow rate between 166.6 and 833.3 mm^3^/s. The theoretical predictions demonstrated that the best results in terms of HI conversion were 74.5% for the methanation membrane reactor and 67% for the simple membrane reactor.

## 1. Introduction

With the simultaneous increase in world energy demand and pressing concerns for reducing greenhouse gases (GHGs) emissions, today, hydrogen has become the most important energy vector. As a result of fast world industrialization, the GHGs emissions resulted are responsible for the climate change, global warming, ocean acidification, and other environmental issues [1]. Carbon dioxide (CO_2_), methane (CH_4_), perfluorinated compounds (PFCs), and nitrogen dioxide (NO) are the main components of GHGs, even though CO_2_ represents the greatest portion among them. Hence, significant efforts have been made to develop effective CO_2_ capture and sequestration (CCS) systems [2]. Hydrogen is considered as a clean and efficient energy vector, playing an important role coupled with the fuel cells for the world’s and developing nations’ energy crises [3]. The use of hydrogen in an economy powered by renewable energy sources will minimize reliance on fossil fuels. It is critical to establish a long-term strategy for massive hydrogen energy generation using non-fossil fuels. The thermochemical splitting of water utilizing high temperature energy from the sun or nuclear sources is one of the potential approaches to create hydrogen in a sustainable manner [4]. The iodine sulphur (IS) thermochemical cycle has been recognized as one of the most promising pathways for hydrogen generation because of its capacity to create hydrogen from water by nuclear heat, which can be provided by high-temperature nuclear reactors [5,6].

The chemical reactions related to the IS thermochemical cycle process are as follows:

Bunsen reaction (T = 100 °C):

SO_2_(g) + 2H_2_O(l) + I_2_(l) → H_2_SO_4_(aq) + 2HI(aq) (1)

Sulfuric acid decomposition (T = 850 °C):

H_2_SO_4_(g) → H_2_O(g) + SO_2_(g) + 0.5O_2_(g) (2)

Hydrogen iodide decomposition (T = 500 °C):

2HI(g) → H_2_(g) + I_2_(g) (3)

The HI decomposition process (3) is responsible for the production of I_2_ and H_2_. The former represents the major product, while I_2_ is recycled to the Bunsen reaction (1). The equilibrium decomposition ratio of HI, which is calculated from the Gibbs free energy of the components (about 20% at 400 °C), is low in this system. As a result of the low breakdown ratio, the quantity of recycled materials (HI and I_2_) increases, lowering the cycle’s thermal performance. A fruitful approach to favor the HI decomposition reaction could be represented by the utilization of hydrogen perm-selective membrane reactors (MRs), where H_2_ is removed from the reaction side through an inorganic membrane [7,8,9,10]. The MRs technology allows that the hydrogen generation and separation may take place simultaneously under an intensified process, reducing the need for extra equipment and lowering the hydrogen separation/purification expenses. The requirement for a separate hydrogen production plant as well as the energy expenditure for hydrogen compression for transportation may be removed by employing hydrogen generators on-site. To carry out the HI decomposition in a MR, membranes with both high H_2_ permeability and H_2_/HI selectivity, as well as strong heat and corrosion resistance in the process environment, are required. In the specialized literature, metallic and silica-based membranes result to be the most investigated systems for hydrogen generation and purification [11,12,13,14]. As demonstrated by Myagmarjav et al. [14], silica membranes offer several benefits over metallic membranes, such as chemical and mechanical resistance, as they are built up on porous ceramic supports. Furthermore, unlike the metallic membranes, such as palladium, tantalum, etc., silica membranes allow a physical separation process (solid-sate diffusion) without any need of superficial reactions, where the hydrogen molecules are dissociated into atoms and then recombined again. Mesoporous silica nanoparticles have been intensively studied as the available nanomaterial in the last decade due to its many promising advantages, such as its extensive multi-functionality, based on its high specific surface, uniform and tuneable pore size, high pore volume, and facile functionalization [15]. For example, Nwogua et al. [16] performed an experimental campaign adopting a novel nano-porous silica ceramic membrane manufactured through an alternative, dip coating method to separate hydrogen from N_2_, Ar, and CH_4_ at at low-pressure and elevated temperatures. Amanipour et al. [17] synthesized a hydrogen-selective silica nano-composite ceramic membrane by depositing a dense layer composed of SiO_2_ and Al_2_O_3_ on the top of a graded multilayer substrate using co-current chemical vapor deposition (CVD).

Hence, the adoption of silica membranes for hydrogen separation may currently represent a more advantageous choice also to carry out the HI decomposition reaction in MRs. To enhance the hydrogen removal from the reaction side of a MR towards the permeate side, where it is collected, a sweep gas or vacuum is frequently employed in this MR zone, in order to favor the enhancement of the hydrogen permeation driving force across the membrane. An alternative option to increase the hydrogen permeation driving force is to perform a secondary reaction in the permeate side that consumes the permeated hydrogen. Hence, the CO_2_ methanation reaction, described in Equation (4), can be utilized to keep the hydrogen concentration in the permeate side low.

CO_2_ + 4H_2_ → CH_4_ + 2H_2_O    ΔH = −165 KJ/mol (4)

The CO_2_ methanation, also known as the Sabatier reaction, represents—therefore—a fruitful method to mitigate the GHGs and, at the same time, to convert CO_2_ into synthetic natural gas (SNG) [17,18,19]. Internal combustion engines could use SNG directly as a fuel. Nevertheless, the availability of pure hydrogen, which is extremely costly, is the reaction’s primary stumbling block. The synergistic impact of combining the CO_2_ methanation and HI decomposition processes in a MR may improve the performance of both processes. The use of a CO_2_ methanation reaction on the permeate side of the MR would result in continuous hydrogen consumption, allowing for a constant chemical potential difference across the membrane while reducing the need for additional vacuum and/or sweep gas to maintain a constant hydrogen concentration gradient. The operating temperature requested for the CO_2_ methanation is regulated by coupling the processes. The hybrid MR coupling the hydrogen generation via HI decomposition and the CO_2_ methanation to generate SNG will address three key issues: (1) overcoming the equilibrium constraint of the HI decomposition process, (2) aiding CO_2_ mitigation, and (3) maintaining a stable hydrogen permeation driving force across the membrane.

It is widely accepted that the theoretical methods are used to find the optimal operating conditions to carry out whatever chemical process, consequently favoring the cost savings in terms of reduced experimental tests. The computational fluid dynamic (CFD) method is a feasible tool for simulating the gas flow characteristics of an industrial system. Based on a control volume technique, it may be adopted to prototype equipment in chemical engineering, such as reformers and separators [20]. Unlike other theoretical models, CFD modeling enables the theoretical visualization of local fluid changes, as well as thermal and mass transport properties. Some of the authors of this manuscript used CFD model to analyze several reaction processes carried out in MRs, such as the esterification [21,22] or hydrogen generation reactions [23,24,25], taking into account variables, such as temperature, product concentration, and velocity distributions in both reaction and permeate sides, as a result of mass and heat transfer, as well as flow resistance. In earlier studies, theoretical models were also used to simulate the HI decomposition reaction in conventional [26,27] and MRs [28,29]. In particular, Goswami et al. [28] used a CFD modeling to simulate the HI decomposition reaction in coated wall MRs, demonstrating that the utilization of membrane into a coated wall reactor can enhance the HI decomposition conversion. Tandon et al. [29] developed a non-isothermal mathematical model by combining the reaction kinetics with the microscopic material and energy balance along with the length of the MR used for the HI decomposition reaction. They performed the simulations comparing the developed model with other already existing isothermal models, optimizing the operating and design parameters.

The novelty of this work deals with the development of the CFD method to analyze the HI decomposition reaction performed in the core of a silica MR, coupling the CO_2_ methanation reaction in the permeate side of the methanation membrane reactor (MMR), meanwhile studying the effect of some operating parameters, such as reaction pressure, feed flow and sweep gas flow rates, for achieving the best performance in terms of HI decomposition conversion, as well as hydrogen recovery. Furthermore, a theoretical comparison between the MMR and the equivalent MR (without conversion of hydrogen and CO_2_ into methane in the permeate side) was proposed and discussed.

## 2. Model Development Using CFD Method

**Figure 1:**
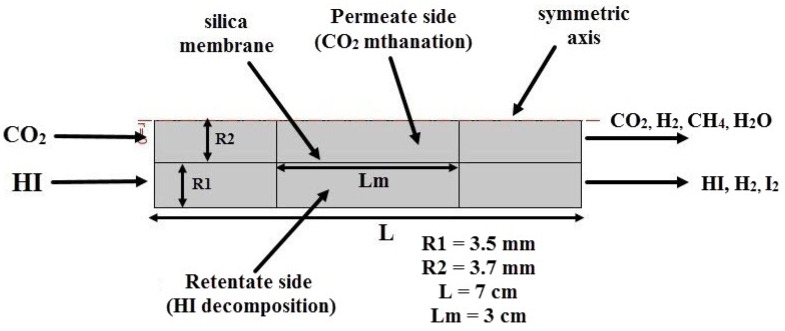
The simplified MR module scheme as a 2D-axisymmetric geometry.

A CFD model was developed using the COMSOL Multiphysics 5.5 software in order to predict the MR module housing a single tubular silica membrane as illustrated in Figure 1, based on the following assumptions:

- Isothermal and steady-state process.
- Consistent membrane and catalytic performance without deactivation or concentration polarization.
- Components with constant physical characteristics.
- Both the retentate and permeate sides have a plug flow pattern.
- In the reaction zone, a pseudo-homogeneous situation is considered.
- At the gas/membrane contact, there is no mass transfer barrier.

According to Figure 1, the “Feed” represents the inlet stream flowing into the silica MR, “Retentate” is the non-permeating outlet stream, and “Permeate” is the stream permeated through the membrane. A 2D axisymmetric model was created using the silica MR-modified shape.

### 2.1. Governing Equations

Continuity equation, Equation (5), momentum balance, Equation (6), and species transport-reaction equation, Equation (7), are the basic expressions used for modeling both permeate and retentate sides of the MR:

(5) $$\nabla \left(\rho_{f}.\varepsilon .u\right)=S_{i}$$

(6) $$\nabla \left(\rho_{f}.u.u\right)=-\nabla p-\beta u+\nabla \tau +\rho_{f}g\;$$

(7) $$\nabla \left(\rho_{f}.u_{i}.\varepsilon \right)=\nabla \left(\rho_{f}D_{i,e}\nabla x_{i}\right)+\left(1-\varepsilon \right)\rho M_{i}\underset{j}{\sum }v_{ij}r_{j}+S_{i}\;$$

where *ρ_f_* is the fluid density, ε is the void fraction of the catalytic bed defined 0.4, *ρ* is the catalyst density, *r_j_* is the reaction rate of components, *v_ij_* is the stoichiometric coefficients, and *M_i_*, *x_i_*, and *D_i,e_* are the molar weight, the mass fraction, and the diffusion coefficient of the component “*i*”, respectively [12]. *β* is the friction coefficient, calculated by Ergun’s equation:

(8) $$\beta =\frac{150\;\mu_{f}{\left(1-\;\varepsilon \right)}^{2}}{\varepsilon^{3}d_{p}^{2}}+\frac{1.75\;\left(1-\varepsilon \right)\rho_{f}}{\varepsilon^{3}d_{p}}\left|u\right|$$

In this equation, *d_p_* is the particle diameter equal to 0.5 mm, ε is the catalyst bed void fraction equal to 0.4, and *μ_f_* is the effective viscosity of the gaseous mixture, which is chosen by the equation proposed by Buddenburg et al. [30]. Moreover, fluid density has been calculated by ideal gas correlation. On the other hand, the Maxwell-Stefan diffusion model was used for calculating the diffusion coefficients.

*S_i_* is the sink/source terms of component “*i*”, which accounts for the addition or removal of the component “*i*” into the system for permeation through the membrane. Here, as a first approximation, hydrogen is considered as the unique gas permeating from the retentate to the permeate side. This term appears as a sink term in the retentate side and a source term in permeate side. In other words, *S_i_* = 0 for all components except for hydrogen, which is calculated as:

(9) $$S_{H_{2}}=\frac{{A\;J}_{H_{2}}M_{H_{2}}}{V}\;$$

A is the membrane surface, V is the computational cell volume, M_H_2__ is the hydrogen molar weight, and J_H_2__ is the hydrogen permeating flux, calculated by Equation (10).

(10) $$J_{H_{2}}={\mathrm{Pe}}_{H_{2}}\left(p_{H_{2},\mathrm{ret}}-{\;p}_{H_{2},\mathrm{perm}}\right)\;$$

Pe_H_2__ (3.8 × 10^−7^ [mol/m^2^/s/Pa]) is the hydrogen permeance for the silica membrane [25], p_H_2_,ret_ is the hydrogen partial pressure in the retentate side, and p_H_2_,perm_ is the hydrogen partial pressure in the permeate side. In Equation (7), *r_j_* is the rate of reaction *j*, and *v_ij_* is the stoichiometric coefficient of component “*i*” in reaction. HI decomposition kinetic reaction on carbon active catalyst may be described according to the equations reported below [13]:

r_HI_ = −kp R_HI_ (11)

(12) $$\;R_{\mathrm{HI}}=\frac{X_{\mathrm{HI}}}{1+K_{I_{2}}{\mathrm{PX}}_{I_{2}}}-\frac{\sqrt{X_{H_{2}}}+X_{I_{2}}\left(1+K_{I_{2}}P\left(\frac{\varphi_{e}}{2}\right)\;\right)}{K_{p}\left(1+K_{I_{2}}{\mathrm{PX}}_{I_{2}}\right)}$$

k = 1.58 × 10^−1^ exp(−E_a1_/RT) (13)

K_I_2__ = 5.086 × 10^−11^ exp(−E_a2_/RT) (14)

where E_a1_ is equal to 34.31 × 10^3^ J/mol and E_a2_ is equal to 86.66 × 10^3^ J/mol, φ_e_ is the equilibrium conversion. The equilibrium constant, K_p_ for the decomposition of HI, is obtained by the free energy values given in the JANAF, using Equation (10).

K_p_ = exp(−∆G(11.5 kJ/mol at 923 °C)/RT) (15)

CO_2_ methanation kinetic reaction on Pt/Al_2_O_3_ catalyst at the permeate side may be expressed according to the equations reported below [12]:

(16) $$r_{m}=\rho_{\mathrm{cat}}\frac{{\mathrm{kK}}_{{\mathrm{CO}}_{2}}K_{H_{2}}^{4}P_{{\mathrm{CO}}_{2}}P_{H_{2}}^{4}}{{\left(1+K_{{\mathrm{CO}}_{2}}P_{{\mathrm{CO}}_{2}}+K_{H_{2}}P_{H_{2}}\right)}^{5}}\left(1-\beta \right)\;$$

(17) $$\beta =\frac{P_{{\mathrm{CH}}_{4}}P_{H_{2}O}^{2}}{P_{{\mathrm{CO}}_{2}}P_{H_{2}}^{4}K_{\mathrm{eq}}}\;$$

k = 1.0635 × 10^11^ exp(−113,497.4/RT) (18)

K_CO_2__ = 9.099 × 10^−7^ exp(69,691.8/RT) (19)

K_H_2__ = 9.6104 × 10^−4^ exp(39,942.0/RT) (20)

K_eq_ = exp((28,183/T^2^ + 17,430/T−8.2536log(T) + 2.8032 × 10^−3^T) + 33.165) (21)

where T is the reaction temperature and R is the ideal gas constant.

### 2.2. Boundary Conditions and Solving Method

The boundary conditions used in the simulations for both permeate and retentate streams are summarized in Table 1. The key parameters (HI conversion and hydrogen recovery), helpful for assessing the silica MR performance during the HI decomposition reaction, are defined in the following equations:

(22) $$\mathrm{HI}\;\mathrm{conversion}\;\left(\%\right)=\frac{{\mathrm{HI}}_{\mathrm{in}}-{\;\mathrm{HI}}_{\mathrm{out}}}{{\mathrm{HI}}_{\mathrm{in}}}*100\;$$

(23) $$\mathrm{Hydrogen}\;\mathrm{recovery}\;\left(\%\right)=\frac{{H_{2}}_{\mathrm{Permeate}}}{{H_{2}}_{\mathrm{Permeate}}+{H_{2}}_{\mathrm{retentate}}}*100\;$$

where HI_in_ and HI_out_ represent the inlet and outlet hydrogen iodide molar flow rates, respectively, and H_2_retentate__ and H_2_permeate__ represent the hydrogen molar flow rates in the retentate and permeate streams, respectively.

As a simulator and to solve the governing equations, the commercial CFD program COMSOL Multiphysics 5.5 was utilized. The finite element approach was utilized to solve the defined two-dimensional CFD model in this program. Pressure-velocity correction has also been conducted using the SIMPLE technique. Standard definitions incorporated in the COMSOL program were used to examine the fluid properties’ dependence on temperature, pressure, and composition over the CFD model domain. The numerical solution was continued until all of the variable’s tolerance values were less than 10^−4^.

### 2.3. Mesh Independency

**Figure 2:**
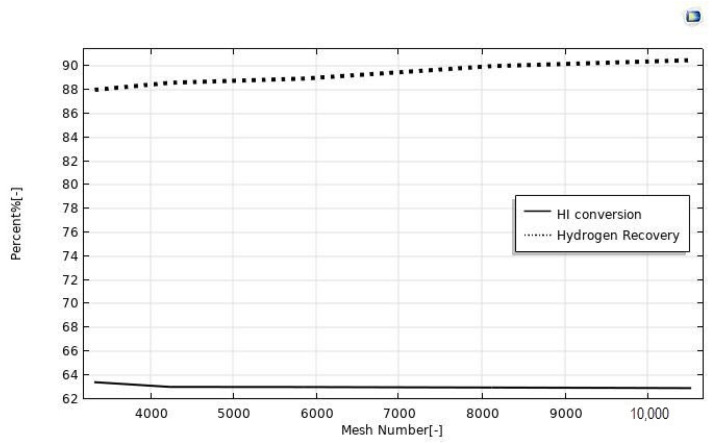
HI conversion and hydrogen recovery as a function of the mesh number. CFD simulations made at *p* = 1 bar, T = 400 °C, sweep gas flow rate = 333.33 mm^3^/s, and feed flow rate = 20 mm^3^/s.

The CFD modeling was carried out considering, at first, various mesh numbers in order to find the optimal value to achieve reasonable results with the lowest solving time. In literature, various criteria were used to optimize the choice of the mesh number, in order to ensure the validity of the simulation results [31,32]. In our case, as already validated in our previous publications [21,22,23,24], we followed an approach to establish the optimized mesh number as a function of a convergence loop. In this regard, the mesh number was optimized when the discrepancies between simulated HI conversion and hydrogen recovery as a function of the mesh number were less than 3%, and this happened in the mesh number range between 3325 and 10,527, Figure 2. Furthermore, beyond the mesh number of 8000, the simulated values became independent of the mesh number itself, owing to insignificant changes. Consequently, a mesh number of 8000 was set in all the simulations of this work.

## 3. Results and Discussion

### 3.1. Model Validation

**Figure 3:**
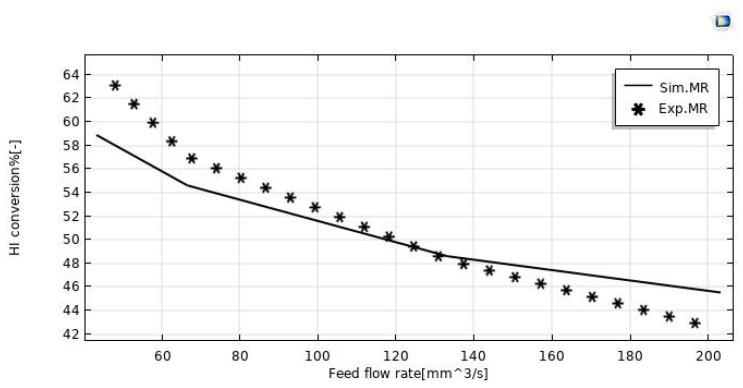
HI conversion in the MR vs. feed flow rate at T = 400 °C, *p* = 1 bar, and sweep gas flow rate = 333.33 mm^3^/s.

The model validation was done in terms of HI conversion to explain the behavior of the silica MR. The validity of the model outputs was confirmed in this case by experimental data taken from Myagmarjav et al. [25]. In this regard, Figure 3 shows both the experimental and modeling results of the HI conversion variations as a function of the feed flow rate during the HI decomposition reaction in the silica MR operated at 400 °C, with 1 bar and sweep gas flow rate equal to 333.33 mm^3^/s. By matching the numerical and experimental data, it was detected an error variation between 7% and 9%. The discrepancies were observed particularly for feed flow rates below 80 mm^3^/s and above 160 mm^3^/s.

### 3.2. Influence of the Reaction Pressure on Component Distributions

**Figure 4:**
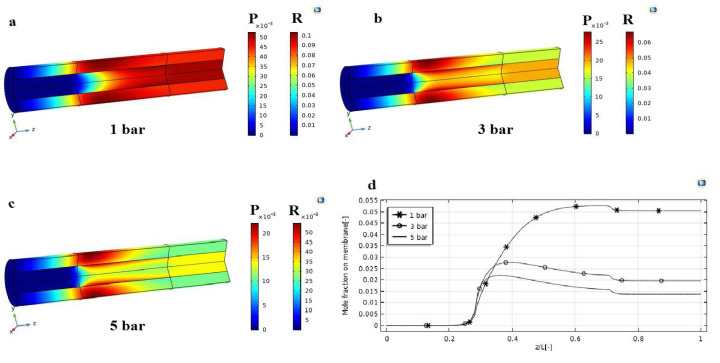
Distributions of hydrogen mole fraction profile in the MMR at T = 400 °C, feed flow rate = 20 mm^3^/s, and sweep gas flow rate = 333.33 mm^3^/s: (**a**) 1 bar; (**b**) 3 bar; (**c**) 5 bar; (**d**) hydrogen mole fraction in the MMR permeate side as a function of z/L.

During the HI decomposition reaction, an important analysis on the effects of the pressure difference (1, 3, and 5 bar) on the concentration contours of hydrogen in MMR was theoretically performed, as shown in Figure 4. In particular, the hydrogen molar fraction contours were analyzed in both axial and radial directions, as shown in Figure 4a–c. It is evident that, also at 1 bar as the pressure gradient, the hydrogen concentration in the retentate side is always above that in the permeate side along the *z*-axis, confirming that the driving force (expressed by the difference of hydrogen partial pressure across the membrane) acts continuously to guarantee the efficient permeation of hydrogen. This is responsible for a raise of the hydrogen molar fraction in the permeate side, as shown in Figure 4a. At a higher pressure difference, the hydrogen permeation driving force is enhanced, favoring a larger removal of hydrogen from the reaction towards the permeate side. This is responsible for the shift of the HI decomposition reaction towards the products, allowing consequently higher conversions. Figure 4b,c showed the change of the hydrogen mole fraction in z and r directions, both in the retentate and permeate sides. At lower pressure in the retentate side, where the HI decomposition reaction takes place, hydrogen is poorly removed from the reaction towards the permeate side due to a reduced driving force. This is why the profile of the hydrogen concentration, shown in Figure 4a, looks quite uniform with a slow reduction along the *z*-axis in both sides. Consequently, the variation of the radial hydrogen concentration is smooth. On the other hand, the higher the pressure, the higher the hydrogen concentration gradient along the *z*-axis, because hydrogen is removed as it is produced, Figure 4d. Hence, the hydrogen concentration in the permeate side is higher near the membrane interface; then, it decreases radially as soon as it reacts with CO_2_ into CH_4_. Along the z direction, the radial profile becomes smoother, achieving a constant gradient due the constant hydrogen permeation driving force.

**Figure 5:**
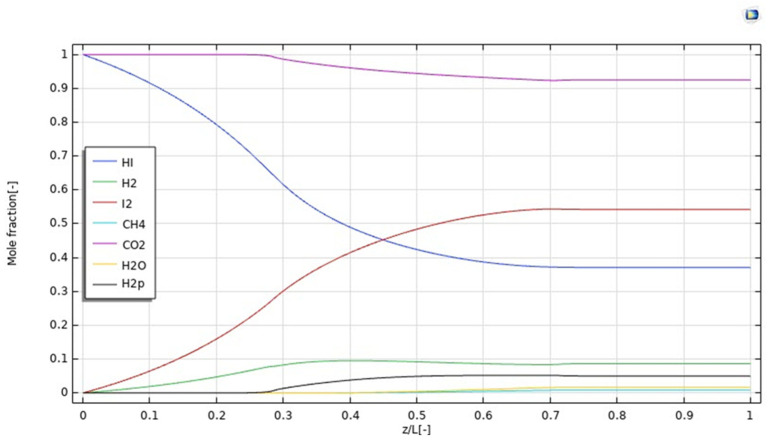
Reactants and products distributions along *z*-axis in the MMR at T = 400 °C, 1 bar, feed flow rate = 20 mm^3^/s, and sweep gas flow rate = 333.33 mm^3^/s.

Furthermore, Figure 5 illustrates the simulation of the molar fraction distribution of reactants and products along the *z*-axis at 400 °C, 1 bar, 20 mm^3^/s as the feed flow rate, and 333.33 mm^3^/s as the sweep gas flow rate. In particular, it is worth of noting that all the molar fractions assume a constant value at z/L > 0.7.

**Figure 6:**
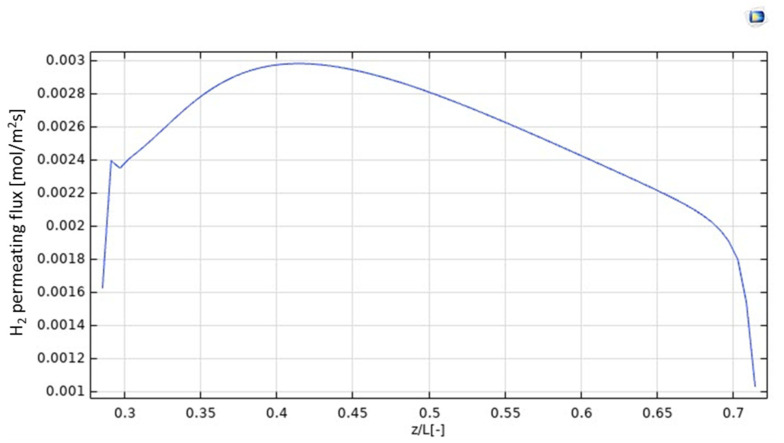
H_2_ permeating flux distribution along *z* axis in the MMR at T = 400 °C and 1 bar.

In Figure 6, the H_2_ permeating flux is plotted as a function of z/L. It increases up to z = 0.4, according to the increasing trend of H_2_ production due to the HI decomposition reaction, which is particularly relevant in the first part of the MMR, as evidenced by the pronounced decreasing trend of the HI molar fraction in Figure 5. In this part of the MMR, the H_2_ permeation driving force is hence high because no H_2_ is present in the permeate side and, as soon as it is collected, it is converted with CO_2_ into CH_4_, due to the methanation reaction. At 0.4 < z/L < 0.7, the molar fraction of HI assumes a slight decreasing trend, meaning that the generation of H_2_ is not consistent, such as at z/L < 0.4. Therefore, as the molar fractions trends of H_2_ in the retentate and permeate sides, and the unconverted CO_2_ and formed CH_4_ in the permeate side become constant, the H_2_ permeating flux slightly decreases, accordingly. Nevertheless, at z/L > 0.7 the molar fraction trend of HI in the retentate side becomes constant, meaning the further H_2_ is not produced and, then, the H_2_ permeating flux drops down rapidly.

### 3.3. Assessment of Operating Parameters Effects

The performance of both the MMR and MR were investigated in terms of HI conversion and hydrogen recovery under various operating conditions, analyzing the effects of the reaction pressure and feed and sweep gas flow rate variations, as reported in Table 2.

#### 3.3.1. Effect of Feed Flow Rate

**Figure 7:**
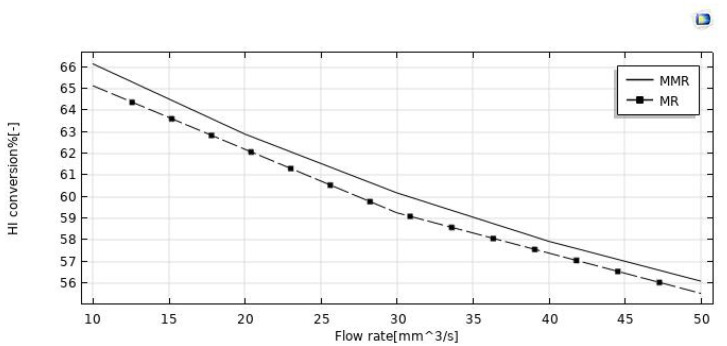
HI conversion vs. the feed flow rate in both the MR and MMR at *p* = 1 bar, T = 400 °C, and sweep gas flow rate = 333.33 mm^3^/s.

The effect of the feed flow rate on the HI conversion was theoretically evaluated at T = 400 °C, reaction pressure = 1 bar, sweep gas flow rate = 333.33 mm^3^/s, and the modeling results between the MR and MMR were compared in Figure 5. By increasing the feed flow rate, HI conversion decreased in both MR configurations, as a consequence of a decreased contact time between the reactants and the catalyst (higher space velocity). Comparing the HI conversions of the two MR configurations, the MR demonstrated lower values in the whole feed flow rates range investigated. Indeed, the hydrogen removed for permeation through the membrane in the MMR was partially transformed in the CO_2_ methanation reaction, resulting in a hydrogen permeation driving force higher than that of the MR. Figure 7 shows the hydrogen recovery as a function of the feed flow rate for both the MR and the MMR. It decreased from ~95.5% to 76% in the MMR and from 96% to 83.5% in the MR. The lower hydrogen recovery values in the MMR with respect to the MR is due to the permeated hydrogen consumption in the MMR permeate side during the CO_2_ methanation reaction.

#### 3.3.2. Effect of Sweep Gas Flow Rate

**Figure 8:**
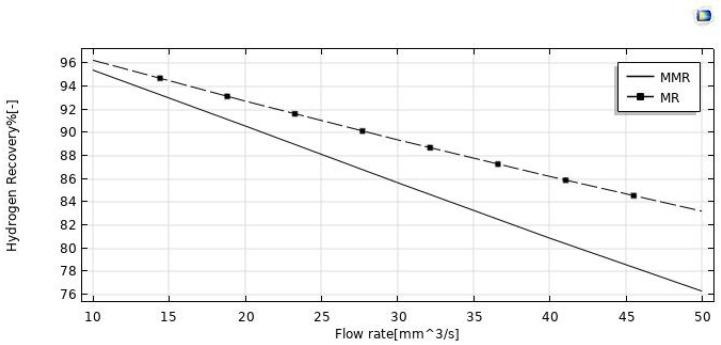
Hydrogen recovery vs. feed flow rate for both the MMR and the MR at *p* = 1 bar, T = 400 °C, and sweep gas flow rate = 333.33 mm^3^/s.

**Figure 9:**
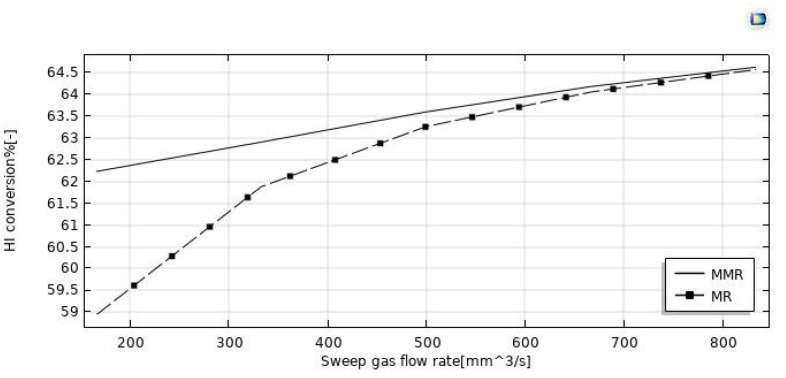
Effect of feed flow rate on the HI conversion at *p* = 1 bar, T = 400 °C, and feed flow rate = 20 mm^3^/s.

**Figure 10:**
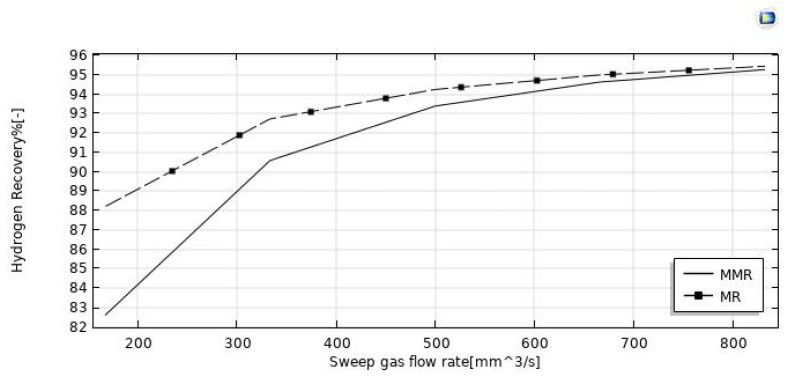
Effect of feed flow rate on the hydrogen recovery at *p* = 1 bar, T = 400 °C, and feed flow rate = 20 mm^3^/s.

The simulations reported in Figure 8 illustrate the increase of HI conversion as the sweep-gas flow rate increases in both the MMR and MR. It may be attributed to the effect of the H_2_ removal from the reaction side towards the permeate side for permeation through the membrane. Indeed, by increasing the sweep gas flow rate, HI conversion and hydrogen recovery (Figure 9 and Figure 10) were enhanced. It may be attributed to the depletion of the hydrogen partial pressure in the permeate side. This induced an increment of the hydrogen partial pressure difference across the membrane, enhancing the hydrogen permeating flux. Meanwhile, it allowed a larger amount of hydrogen to be collected in the permeate side, with a consequently more pronounced shift effect on the HI decomposition reaction (higher conversions) and a higher hydrogen recovery. In the MMR, the depletion of the hydrogen concentration in the permeate side was greater due to the utilization of hydrogen during the CO_2_ methanation reaction. This involved a higher hydrogen permeation driving force than that present in the MR, and consequently higher conversions were reached. Nevertheless, at a sweep gas flow rate superior to 700 mm^3^/s, no difference was observed between the MMR and MR systems because the depletion of the hydrogen concentration in the permeate side became irrelevant. Obtaining better performance by increasing the sweep gas flow rate is not very attractive, because higher sweep gas flow rates mean higher costs.

#### 3.3.3. Effect of Reaction Pressure

**Figure 11:**
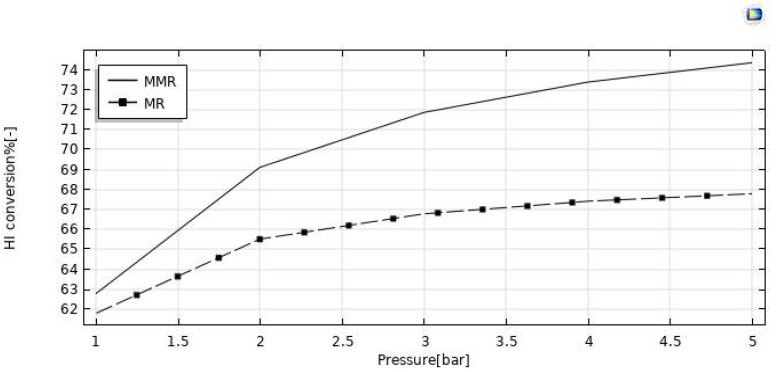
HI conversion vs. reaction pressure at T = 400 °C, feed flow rate = 20 mm^3^/s, and sweep gas flow rate = 333.33 mm^3^/s.

**Figure 12:**
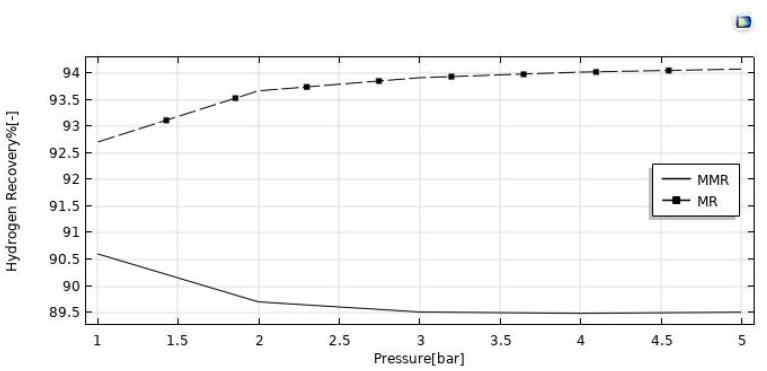
Hydrogen recovery vs. reaction pressure at T = 400 °C, feed flow rate = 20 mm^3^/s, and sweep gas flow rate = 333.33 mm^3^/s.

The reaction pressure effects on the HI conversion and hydrogen recovery are illustrated in Figure 11 and Figure 12. By increasing the retentate pressure, the driving force of the hydrogen permeation through the membrane is enhanced for both the reactors, but the hydrogen recovery demonstrated opposite trends as a function of the reaction pressure in the MR (increasing) and in the MMR (reducing). When removing hydrogen from the reaction zone for the Le Chatelier’s principle, the forward reaction of HI decomposition is thermodynamically favored, and this led to higher conversions of HI. On the other hand, the conversion in the MMR was higher than that in the MR due to an enhanced hydrogen permeation driving force, caused by a lower hydrogen partial pressure in the permeate side determined by the hydrogen consumed in the CO_2_ methanation reaction. The same reason can be considered for the hydrogen recovery, which was enhanced in the MMR as a consequence of a pressure increase, whereas in the MR the detrimental effect due to a pressure increase was not completely counterbalanced by the “shift effect” related to the membrane permeation, causing a decreasing trend globally.

#### 3.3.4. Optimized Operating Conditions

The analyses on the performance of both the MR and MMR systems evidenced that the best results were obtained considering—as main guidelines—the achievement of the best compromise within the highest hydrogen recovery and the highest HI conversion. For the MMR system, the main objective was to favor the highest transformation of CO_2_ into SNG in the MMR system. In this regard, a relevant role was played by the recovery of hydrogen produced during the HI decomposition, which—permeating through the membrane—reacts with CO_2_ under the methanation reaction to produce SNG. Therefore, Table 3 illustrates that the optimized operating conditions able to allow the best performance for the MMR system, 66% of the HI conversion and 95.5% of the hydrogen recovery, were 400 °C, 1 bar, 10 mm^3^/s of the feed flow rate, and 333 mm^3^/s of the sweep-gas flow rate. On the other hand, for the MR system, the best compromise within HI conversion (65%) and hydrogen recovery (93.6%) was reached at 400 °C, 2 bar, 20 mm^3^/s of feed flow rate, and 333 mm^3^/s of sweep-gas flow rate.

## 4. Conclusions

A CFD model was developed and validated with experimental results for studying and understanding the performance of silica MRs in an HI decomposition reaction in depth. Based on the viable equations included in the MR model, it was observed that an increase in the feed flow rate from 10 mm^3^/s to 50 mm^3^/s led to a significant HI conversion decrease from 66% to 56% in the MMR and from 65% to 55.5% in the MR. On the other hand, the hydrogen recovery decreased from 95.5% to 76% in the MMR and from 96% to 83.5% in the MR. On the other hand, an increase of pressure from 1 to 5 bar determined an increase of HI conversion from 63% to 74% in the MMR and from 62% to 68% in the MR, while the hydrogen recovery remained substantially constant around 90% in the MMR, and a slight increase from 92.5% to 94% was reached in the MR. Globally, the simulations demonstrated better performance achievable in the MMR than in the MR under all the experimental conditions investigated, with the further advantage of transforming CO_2_ into SNG. Furthermore, the excellent performance in terms of HI conversion and hydrogen recovery in both the MMR and MR proved the high potential application of silica membranes in MRs for the simultaneous hydrogen generation and purification.

## Figures and Tables

**Table 1:** Boundary condition at retentate and permeate sides.

Position	Retentate Side	Permeate Side
Z = 0	inflow	Inflow
Z = L	outflow	Outflow
r = R_1_	flux	Flux
r = R_2_	$\frac{\partial c}{\partial r}=0$	$\frac{\partial c}{\partial r}=0$

**Table 2:** The investigated operating conditions for parametric analyses of the MMR and MR performance during the HI decomposition reaction.

Operating Parameters	Pressure	Feed Flow Rate	Sweep Factor
Temperature (°C)	400	400	400
Pressure (bar)	**1–5**	1	1
Feed flow rate (mm^3^/s)	20	**10–50**	20
Sweep gas flow rate (mm^3^/s)	333.33	6.22	**166.6–833.3**

**Table 3:** Optimized operating condition to reach the best performance in the MR and MMR systems.

		MMR				MR	
Operating Parameters		HI Conversion [%]	H_2_ Recovery [%]	Operating Parameters		HI Conversion [%]	H_2_ Recovery [%]
Temperature (°C)	400	66	95.5	Temperature (°C)	400	65	93.6
Pressure (bar)	1			Pressure (bar)	2		
Feed flow rate (mm^3^/s)	10			Feed flow rate (mm^3^/s)	20		
Sweep gas flow rate (mm^3^/s)	333			Sweep gas flow rate (mm^3^/s)	333		

## Data Availability

Not applicable.
